# RNA-seq-based analysis of the hypertrophic scarring with and without pressure therapy in a Bama minipig model

**DOI:** 10.1038/s41598-018-29840-6

**Published:** 2018-08-07

**Authors:** Baimei Liu, Yang Liu, Li Wang, Chunsheng Hou, Meiwen An

**Affiliations:** 1 Institute of Applied Mechanics and Biomedical Engineering, Taiyuan University of Technology, Taiyuan, 030024 China; 2 Shanxi Key Laboratory of Material Strength & Structural Impact, College of Mechanics, Taiyuan University of Technology, Taiyuan, 030024 China; 3 National Demonstration Center for Experimental Mechanics Education (Taiyuan University of Technology), Taiyuan, 030024 China; 4 Department of Burns and Plastic Surgery, Taigang General Hospital, Taiyuan, 030009 China

## Abstract

Pressure therapy has been proved to be an effective treatment for hypertrophic scars in a clinical setting. However, evidence-based data are controversial and the precise mechanism of action of this technique remains unknown. The aim of this study was to investigate the potential molecular mechanisms of pressure therapy for hypertrophic scars. We established a Bama minipig (*Sus scrofa*) model of hypertrophic scarring in which the scars were treated with pressure to explore the mechanism of action of the treatment. There were 568 differentially expressed genes (289 upregulated, 279 downregulated) after pressure therapy at 90 days post-injury, whereas only 365 genes were differentially expressed (250 upregulated, 115 downregulated) at 120 days post-injury. These genes were associated with metabolic pathways, ECM-receptor interaction, the PI3K-Akt and MAPK signaling pathways, focal adhesion and cytokine-cytokine receptor interaction. In addition, the qRT-PCR results indicated that the trend of gene expression following pressure therapy was mostly consistent across the two methods. In conclusion, our systematic analysis of the transcriptome has provided a better understanding of the molecular mechanisms involved in pressure therapy and offers an important basis for further studies of the complex signaling pathways regulated by the treatment.

## Introduction

Hypertrophic scarring (HS) is a common form of pathological injury following cutaneous wounding and leads to severe esthetic and functional disfigurement in affected patients^1–4^. The pathophysiology of the underlying process of scar formation remains unknown. Pressure therapy is generally accepted as one of the best noninvasive techniques for preventing and controlling hypertrophic scarring after burn injury^5,6^. The rational use of pressure can reduce collagen production, alleviate pain and itchiness, and tends to prevent the development of serious contractures^7–9^. Although this is an efficient method, the mechanisms responsible for hypertrophic scarring remission following pressure therapy are not well understood. Therefore, a better understanding of the mechanisms underlying the pressure treatment may result in improved clinical effectiveness.

Hypertrophic scar formation is a complex biological process under the strict control of specific genes^10^. The mechanisms of pressure therapy for hypertrophic scarring have been investigated in humans and animal models^11–13^. Numerous studies have demonstrated that mechanical stress can affect cell proliferation and apoptosis by activating cellular signaling pathways^14,15^. Pressure therapy that suppresses scar hyperplasia also results in tissue ischemia. Scar hyperplasia is restricted when the pressure reaches 1.33–2.00 kPa^16^. In hypoxic environments mitochondria become swollen and vacuolated causing fibroblasts to be inhibited and their ability to synthesize collagen fibers reduced. Ischemia can result in reduced alpha-macroglobulin and increased collagenase concentrations, leading to damaged collagen fibers^17^. The deposition and synthesis of mucopolysaccharides reduces following the decrease in enzyme concentration, resulting in decreased collagen generation and reduced scarring. However, these studies do not completely reflect the complex biological processes caused by pressure treatment.

The Bama minipig is a mammal in which the appearance of scars, their histological aspects, biochemistry, immunology, molecular biology and clinical behavior are very similar to those in humans^18,19^. In previous study, we established a model of hypertrophic scarring in this pig and applied pressure treatment at 60 days post injury. Gross changes in the general appearance and size of the wounds were observed at different time points. At 14 days post-injury, all wounds were completely filled with granulation tissue, and partial re-epithelialization was observed at the wound margins. Beginning at day 30 and continuing to the end of the study, all wounds were observed to have a dense, fibrotic appearance, comparable to that previously reported in skin wounds on female red Duroc pigs^20^. The scar at 60 days was within the original margin range, clearly tougher than the surrounding normal skin, appearing deep red in the center. Furthermore, in comparison to the untreated group, the pressure-treated group exhibited a pigmented area that was smaller, with less contraction and some softening of the scar surface, and the size of wounds was smaller. Moreover, The resulting scars were histopathologically identical to human hypertrophic scars^21^. HE and Masson staining results showed that the connective tissues were thinner after one month pressure intervention, and the collagen bundles were regular and parallel to the surface as observing in normal skin after two months pressure intervention. Thus, in this study an *in vivo* model based on this animal was used to observe changes in the transcriptome with and without pressure therapy.

Transcriptomics is a technique used to survey changes in RNA expression at a given moment in time^22^. Transcriptome sequencing technologies are the foundation for transcriptomic studies^23^. RNA-Seq has been demonstrated to be an effective approach for transcriptome analysis, which provides global, integrated and specified information about a tissue^24^. Based on deep sequencing, RNA-Seq has been widely used to investigate the pathogenesis of many complex diseases and a variety of other fields, due to its high-throughput and low cost. Previous studies have shown that increased levels of growth factors and extracellular matrix (ECM) production secreted by fibroblasts play key roles in the formation of hypertrophic scars^25–27^. However, changes in the transcriptome during the formation of scar and pressure treatment has not yet been reported.

Therefore, in this study, a Bama minipig model of hypertrophic scarring was established, with pressure applied to dermal injuries to explore the underlying mechanism of this therapy. We used RNA-Seq to evaluate the effects of pressure treatment on gene expression in samples of the scar at various time points. This study contributes to the understanding of the molecular mechanisms and signaling pathways through which pressure affects hypertrophic scar development and regression.

## Results

### RNA-Seq quality assessment

After stringent quality assessment of sequencing, more than 33 million total original reads for each sample were obtained, the proportion of bases with quality values greater than 20 (Q20) was more than 96%. These results indicated that the quality of the sequencing results was acceptable (Table 1). After filtering out the adaptor sequence and low quality reads, the percentage of clean reads within the raw reads accounted for 94% of the total sequences in four groups. The high proportion of clean reads and low number of low-quality or adaptor sequences demonstrated the excellent quality of the sequencing, which laid the foundation for high quality subsequent information analysis. Tophat software was used to map the obtained clean reads to a pig (*sus scrofa*) reference genome (National Center for Biotechnology Information reference sequence: GCA_000003025.6). As shown in Table 1, approximately 77.2% of the clean reads were mapped onto the reference genome.

**Table 1:** Output statistics of sequencing and mapping. Clean ratio = (Clean reads/Raw reads) × 100%.

Sample ID	Q20 Value (%)	Raw reads	Clean reads	Clean ratio	Mapped reads	Mapped Unique reads	Mapping ratio
90d	97.1	35,165,052	33,526,198	95.40%	26,290,851	24,802,330	78.3%
90d + P	96.6	34,565,854	32,780,576	94.84%	25,862,635	24,748,103	78.9%
120d	96.9	37,213,613	35,371,718	95.06%	27,390,731	26,329,848	77.5%
120d + P	96.4	37,319,865	35,371,659	94.78%	26,376,327	25,166,477	74.3%

### Identification of differentially expressed genes (DEGs)

**Figure 1:**
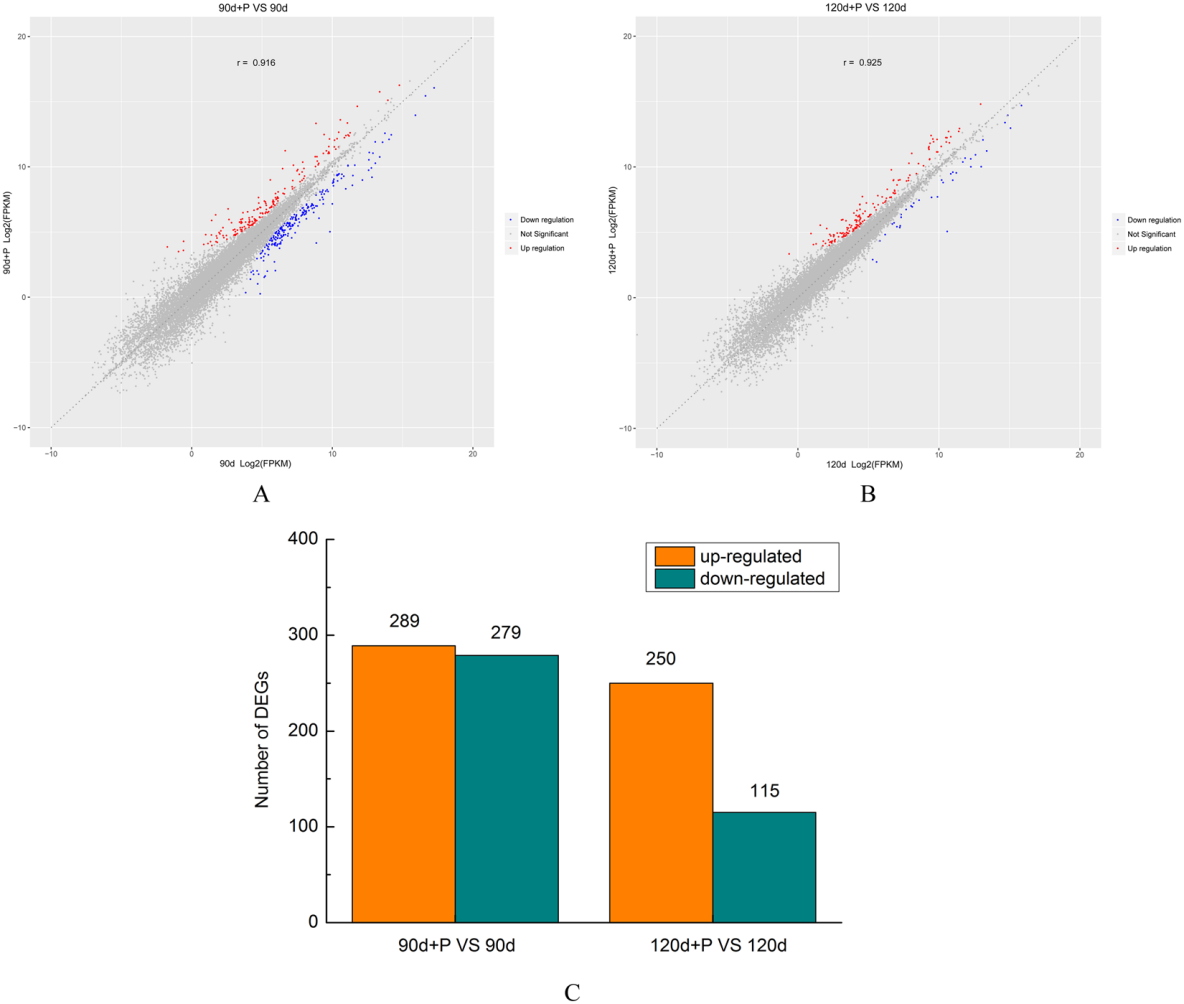
Differentially expressed genes of the hypertrophic scar (HS) tissues before and after pressure treatment at 90 days and 120 days post injury. The vertical coordinates represent the log2(FPKM) values for each gene in the HS treated with pressure, and the horizontal coordinates represent the log2-FPKM values for each gene in the HS without treatment. Red, upregulated genes; blue, downregulated genes; gray, unchanged gene. (**A**) Samples at 90 days post injury; (**B**) samples at 120 days post injury; (**C**) histogram of the numbers of differentially expressed genes in the two groups (FDR ≥1).

Changes in the transcriptome expression levels caused by pressure treatment may reveal the biological effect of therapeutic approaches to HS formation and progression. Transcriptional changes resulting from the pressure treatment were evaluated using genome-wide transcriptome profiling of HS tissues, via RNA sequencing. The results of differential gene expression analysis are shown in Fig. 1. According to the screening conditions for differentially expressed genes (p-value ≤ 0.05, fold-change ≥2), 568 genes were markedly differentially expressed in the HS tissue treated with pressure compared with the untreated group at 90 days post-injury. Of these, the expression of 289 genes (50.9%) was up-regulated and down-regulated in 279 (49.1%). In contrast, a total of 365 genes were detected that were significantly differentially expressed in HS treated with pressure compared with the untreated group at 120 days post-injury. Of these, 250 genes (68.5%) were up-regulated and 115 (31.5%) were down-regulated (Fig. 1C). The number of DEGs was smaller when pressure therapy was used for two months compared to those where treatment was for only one month. These gene expression patterns illustrated the presence of a diverse and complex regulatory system in HS.

### Gene Ontology (GO) Analysis

**Figure 2:**
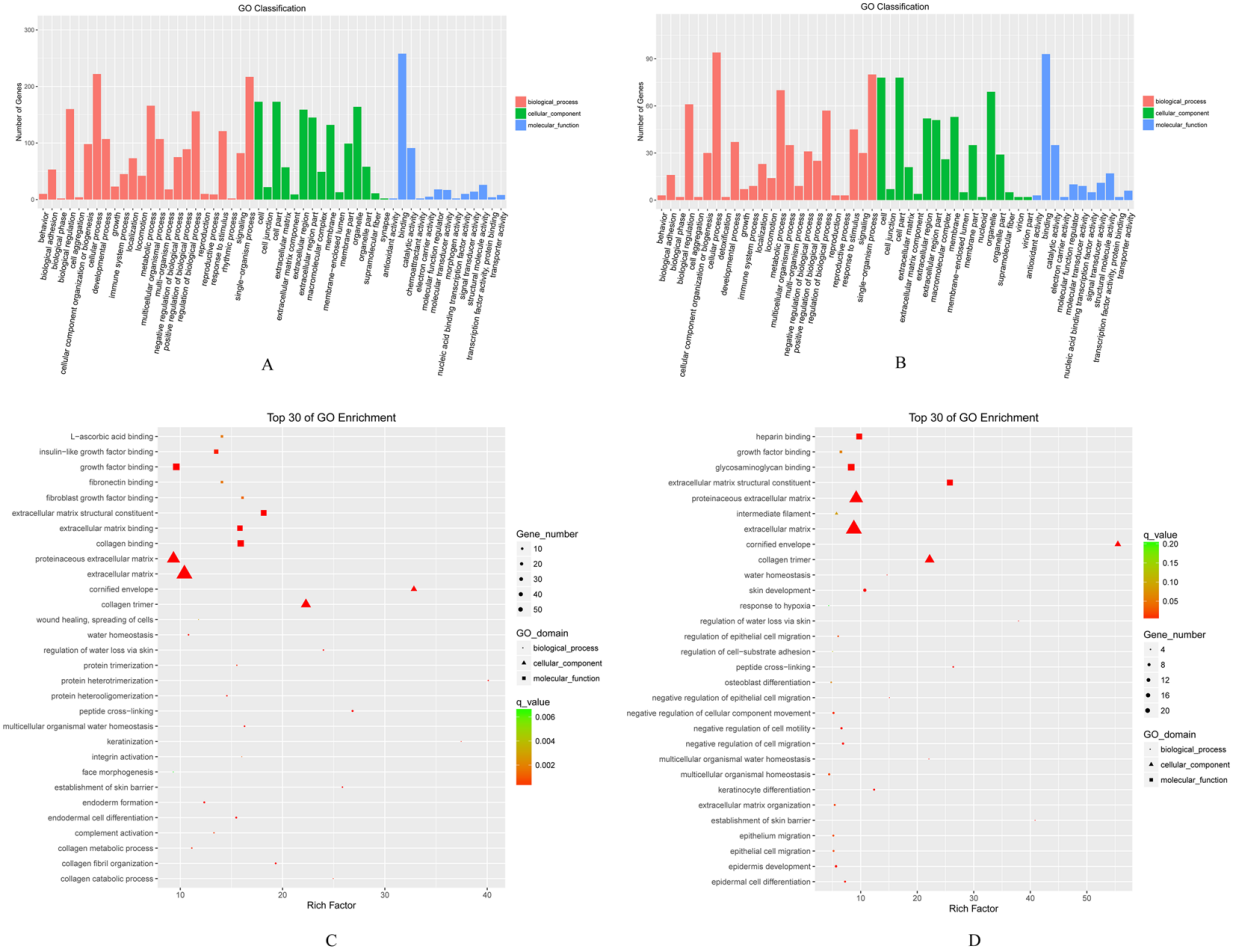
Gene Ontology (GO) analysis. The significant GO category for differentially expressed genes in the levels of biological process, cellular component and molecular function at at day 90 (**A**), day 120 (**B**) post injury, compared with untreated hypertrophic scar. The top 30 of GO terms enrichment of 90 days group and 120 days group were shown in (**C**) and (**D**) respectively.

In order to further clarify the functions of all differentially expressed genes (DEGs), GO functional classification for the DEGs of ‘90d + P’ vs. ‘90d’ and ‘120d + P’ vs. ‘120d’ was performed. A total of 633 GO terms were classified into 52 functional categories in the ‘90d + P’ vs. ‘90d’ group, and 238 terms were classified into 52 functional categories in the ‘120d + P’ vs. ‘120d’ group. We enumerated the DEGs in the three GO domains: biological process, cellular component and molecular function. The number of DEGs with GO annotations obtained from the ‘90d + P’ vs. ‘90d’ group samples was greater than those obtained from the comparison of ‘120d + P’ vs. ‘120d’. However, the proportion of DEGs having the same GO term within the two groups was almost same (Fig. 2). Concerning the biological processes domain, which describes the greatest number of DEGs, GO terms enriched with DEGs included cellular process, single-organism process, metabolic process, biological regulation and response to stimulus. Regarding the cellular components, the cell and cell part component were relatively enriched with DEGs. For molecular functions, the GO terms enriched with DEGs included binding, catalytic activity, structural molecular activity, molecular function activity and signal transducer activity. In addition, we performed GO enrichment analysis. The results of the top 30 GO enrichment terms in the two groups are shown in Fig. 2C,D.

### Kyoto Encyclopedia of Genes and Genomes (KEGG) pathway enrichment analysis of DEGs

**Figure 3:**
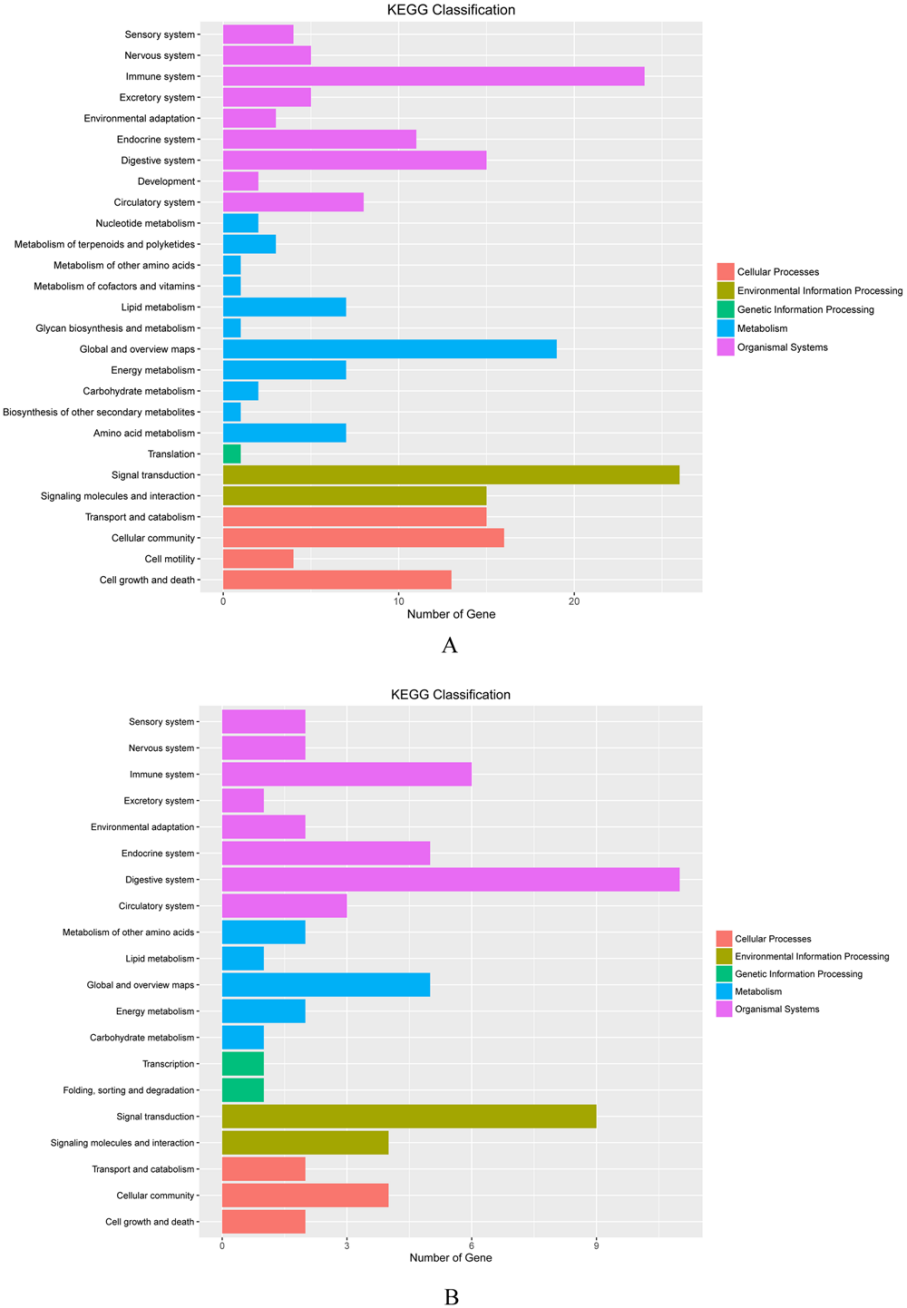
Kyoto Encyclopedia of Genes and Genomes (KEGG) pathway enrichment analysis. The significant pathway for differentially expressed genes at day 90 (**A**) and day 120 (**B**) post injury, compared with untreated hypertrophic scar.

In order to further understand the biological functions of all the DEGs, we performed KEGG pathway classification analysis. In the present study, 313 DEGs had annotations that belonged to 113 KEGG pathways in the ‘90d + P’ vs. ‘90d’ group, and 111 DEGs had annotations to 60 KEGG pathways in the ‘120d + P’ vs. ‘120d’ group. These pathways were classified into 27 and 20 higher level pathways, respectively, belonging to organismal systems, metabolism, genetic information processing, environmental information processing and cellular processes (Fig. 3). Furthermore, we also performed KEGG enrichment analysis. A portion of significantly enriched (p-value ≤ 0.05) pathways of the ‘90d + P’ vs. ‘90d’ and ‘120d + P’ vs. ‘120d’ comparisons were shown in Tables 2 and 3, respectively. The DEGs contained most enrichment were located in metabolic pathways and PI3K-Akt signaling pathway. In addition, DEGs were significantly enriched in the ECM-receptor interaction, focal adhesion and Apoptosis pathway terms for ‘90d + P’ vs. ‘90d’ group, whereas fewer DEGs were enriched in the ‘120d + P’ vs. ‘120d’ group. The top 5 significantly overrepresented pathways in this group were associated with metabolic pathways, the PI3K-Akt signaling pathway, the focal adhesion, ECM-receptor interaction and cytokine-cytokine receptor interaction. These annotations from samples of HS treated with pressure provided a valuable resource for further investigation of the formation and developmental processes of HS and for refining therapy in clinic.

**Table 2:** Pathways significantly differently enriched in hypertrophic scar at 90 days post injury treated with or without pressure.

Pathway	Differential genes	Upregulated genes	Downregulated genes	Total genes
Metabolic pathways	19	17	2	902
PI3K-Akt signaling pathway	11	2	9	214
Focal adhesion	9	1	8	118
ECM-receptor interaction	9	1	8	48
Apoptosis	6	4	2	107
Cytokine-cytokine receptor interaction	5	4	1	176
Chemokine signaling pathway	5	4	1	124
p53 signaling pathway	5	3	2	46
Ras signaling pathway	4	4	0	147
MAPK signaling pathway	2	1	1	155

**Table 3:** Pathways significantly differently enriched in hypertrophic scar at 120 days post injury treated with or without pressure.

Pathway	Differential genes	Upregulated genes	Downregulated genes	Total genes
Protein digestion and absorption	7	7	0	60
Metabolic pathways	5	2	3	902
Alzheimer’s disease	4	4	0	127
PI3K-Akt signaling pathway	3	3	0	214
ECM-receptor interaction	3	3	0	48
Focal adhesion	3	3	0	118
cAMP signaling pathway	3	3	0	128
cGMP-PKG signaling pathway	3	3	0	108
AGE-RAGE signaling pathway in diabetic complications	2	2	0	70
Ras signaling pathway	2	2	0	147

### Cluster analysis of significant differential genes in pressure-treated scars

**Figure 4:**
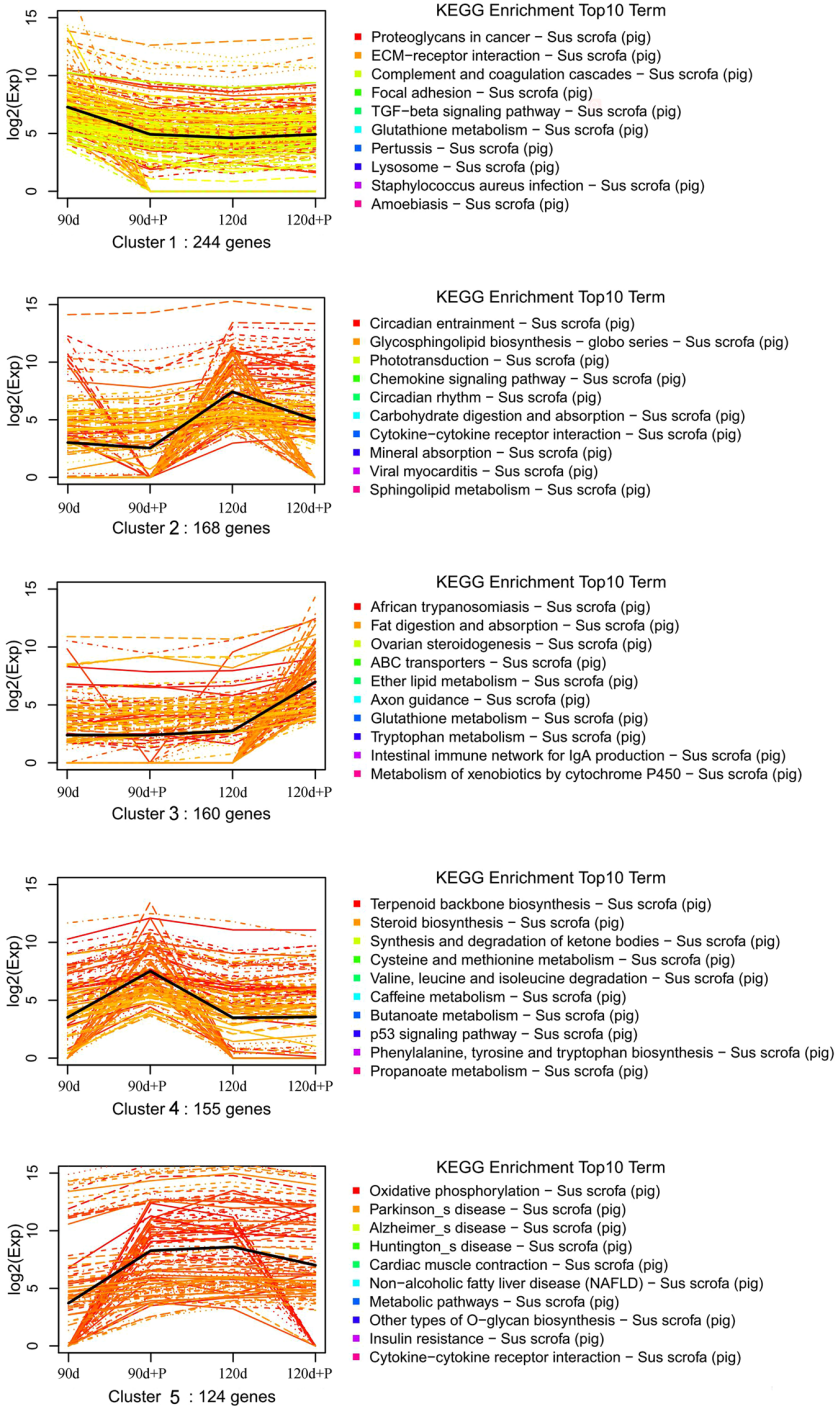
Categories distribution of differentially expressed genes in the top 5 clusters. Clusters were obtained by the K-means method on the gene expression profiles of the 2199 modulated genes. Top 10 Kyoto Encyclopedia of Genes and Genomes (KEGG) enrichment pathways of each cluster were also analyzed.

As is well known, genes with similar expression patterns are usually functionally correlated. In this study, k-means clustering was used to group differentially expressed genes, in accordance with their similarity. After cluster analysis, a total of 1214 genes were classified into 10 profiles based on their expression modulation, each cluster had the similar expression patterns after pressure therapy. Among these profiles, we identified the top 5 profiles of gene expression, containing a total of 851 genes (Fig. 4). Thus, these more significant profiles were considered to have potential to be the main expression profiles in our experiment. The functional GO categories and enriched KEGG pathways were also calculated for each cluster to identify differences in the distribution of genes after different durations of therapy. Cluster 1 contained genes that were negatively modulated after one month of pressure therapy, but which did not alter significantly after two months of treatment. The top 10 enriched GO terms included extracellular matrix and extracellular region genes. The enriched KEGG pathways in this cluster included proteoglycans in cancer and ECM-receptor interaction. Cluster 2 contained genes negatively modulated after two months of pressure therapy, with no significant change in gene expression after one month’ treatment. The top 10 GO terms enriched with these genes included response to leptin and positive regulation of macrophage activation. The most enriched KEGG pathway in this cluster was circadian entrainment. Differentially expressed genes in cluster 3 were not significant after one month of pressure therapy, and then up-regulated after two months’ treatment. The GO terms enriched with these genes consisted of glutathione biosynthetic process and cornified envelope. The most enriched KEGG pathway in this cluster was African trypanosomiasis. The total of 155 genes in cluster 4 were significantly up-regulated after pressure therapy for one month. The remaining gene expression profiles that altered after both one and two months of pressure treatment fell into cluster 5. The top GO terms with these genes were enriched in respiratory chain and NADH dehydrogenase activity and the most enriched KEGG pathway was the oxidative phosphorylation.

### Confirmation of altered gene expression in Hypertrophic Scar (HS) treated with pressure using qRT-PCR

**Figure 5:**
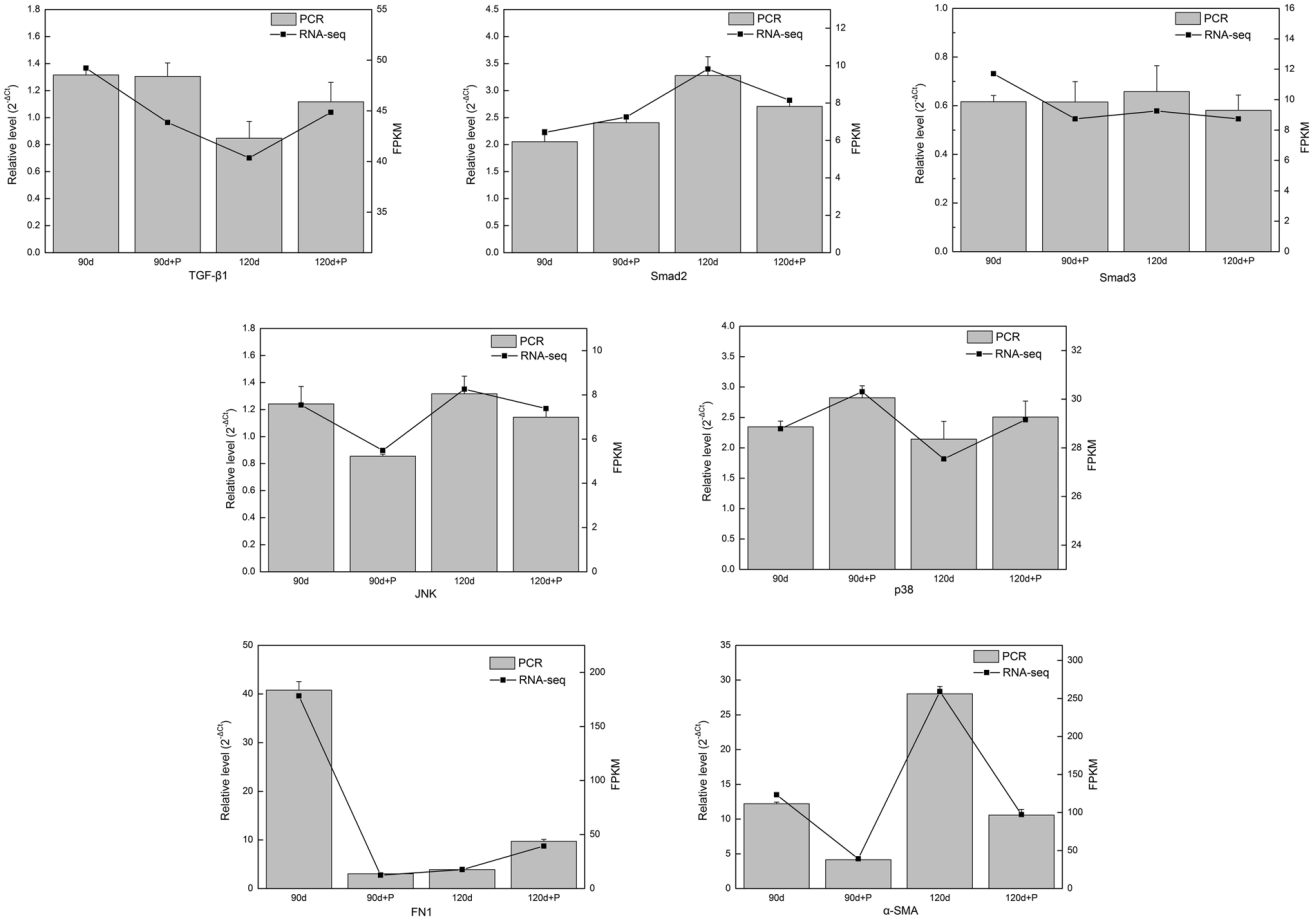
Confirmation of altered expression of genes identified by RNA sequencing (RNA-seq). Seven genes correlated with hypertrophic scar formation and progression following pressure treatment were assayed via qPCR to establish the validity of the RNA sequencing differential expression analysis. FPKM: Fragments Per Kilobase of exon model per Million mapped reads.

In order to confirm the results of the RNA-seq of HS treated with pressure, seven genes associated with HS formation and development were selected for quantitative reverse-transcription polymerase chain reaction (qRT-PCR) analysis. As shown in Fig. 5, expression of SMAD family member 2 (Smad2), Alpha-smooth muscle actin (α-SMA) and fibronectin 1 (FN1) measured by qRT-PCR were in complete agreement with the pattern of RNA-seq analysis. A further two genes including c-jun N-terminal kinase (JNK) and p38 MAP kinase (p38) behaved similarly with only transforming growth factor beta 1 (TGF-β1) and SMAD family member 3 (Smad3) not showing consistent expression behavior in qRT–PCR and RNA-seq analysis at 90 days post-injury. Overall, gene expression levels measured by RNA sequencing and PCR were highly significantly correlated, indicating that RNA-seq expression analysis was reliable and accurate in this study. Gene expression of TGF-β1 and Smad3 were down-regulated in HS treated with pressure at 90 days post-injury compared with the untreated group. However, Smad2 gene expression were up-regulated after one month of pressure treatment. Expression of the Smad2 and Smad3 genes declined in the two months’ pressure-treated group. In addition, the FN1 and α-SMA expression were decreased in the one month’ pressure-treated group, whereas the FN1 expression was up-regulated and the α-SMA was down-regulated after pressure therapy at 120 days post-injury. Furthermore, JNK gene expression was down-regulated and p38 gene expression in HS were up-regulated after pressure treatment compared with untreated groups.

## Discussion

Pressure therapy for hypertrophic scars has proved to be an effective clinical technique through acceleration of fibroblast apoptosis and a reduction in extracellular matrix deposition^11,28^. However, evidence-based data are controversial and the precise mechanism of action of the therapy remains unknown. A global transcriptome analysis can facilitate identification of systemic gene expression and regulatory mechanisms of pressure treatment^29,30^. In the present study, we established a Bama minipig model of hypertrophic scar and used an RNA sequencing method that allowed rapid and efficient deep sequencing to elucidate the global molecular events associated with the mechanisms of pressure therapy. In comparison to the untreated group, the pressure-treated group exhibited a pigmented area that was smaller, with less contraction and some softening of the scar surface after just one month of pressure therapy. Furthermore, compared with the untreated group, HS treated with pressure showed significant changes in their gene expression levels and metabolic pathways.

The transcriptome comprises the sum total of mRNA of genes expressed at a single point in time, and so will change at specific developmental stages or for a particular physiological condition. The ability to analyze the transcriptome is essential for querying functional elements of the genome, revealing the molecular mechanisms of cells and tissues and to understand the development of diseases^31^. RNA-seq has been widely used to both qualitatively and quantitatively analyze transcriptomes due to the technique’s high throughput, accuracy and low cost, and has been used successfully for *de novo* transcriptome sequencing and assembly in many organisms^32,33^. In this study, approximately 33 million sequence raw reads were generated for each sample by Illumina sequencing of the mRNA from the four HS tissues treated with and without pressure. Although the genome of the Bama minipig (*sus scrofa*) examined in this study was not the same as the reference genome, 77.2% reads were successfully mapped onto the known sequence of the pig genome. At the current level of sampling, the sensitivity of the RNA-Seq approach allowed accurate identification of differentially expressed genes (DEGs), their precise quantitative expression, even of those genes that had been weakly transcribed, as confirmed by real-time RT-PCR analysis. Furthermore, our results led to the identification of 568 and 365 DEGs at 90 days and 120 days post-injury, respectively, suggesting that there is considerably more transcript complexity after one month of pressure therapy than two months for HS treatment.

According to the GO functional analysis of our RNA-seq results, these DEGs involved more than 50 functional categories including cellular process, single-organism process, metabolic process and the biological phase terms within the biological processes domain; cell and cell part, organelle, membrane and extracellular region in the cellular component domain; binding, catalytic activity, structural molecular activity, molecular function activity and signal transducer activity in the molecular function domain. We also observed an interesting phenomenon, not only in the 90 days group but also in the 120 days group, where the DEGs were mostly enriched in the same previously described GO functional categories, although the number of DEGs in the 120 days group were fewer than in the 90 days group. This may demonstrate that more genes participate in the process of pressure therapy at the early stage. Furthermore, we found that these GO terms were enriched with insulin-like growth factor 1 binding, extracellular matrix binding, collagen trimer and collagen fibril organization in the 90 day group. This discovery was consistent with the study conducted by Huang and colleagues, who demonstrated that pressure therapy upregulated matrix metalloproteinase expression and downregulated collagen expression in hypertrophic scar tissue^11^. However, in the 120 days group, the GO terms were enriched with glycosaminoglycan binding, extracellular matrix, collagen trimer, and proteinaceous extracellular matrix, suggesting that pressure therapy also affected the activity of glycosaminoglycan and collagen metabolism, which was also found in the Tejiram’s study demonstrating that compression therapy can affect collagen type balance in hypertrophic scar^13^.

Numerous studies have confirmed that a large number of signaling transduction pathways participate in the formation and progression of hypertrophic scars. For example, it has been reported that activation of the PI3K/Akt signaling pathway inhibits the apoptosis of fibroblasts in hypertrophic scars^34–36^. Moreover, the MAPK signaling pathway has been generally investigated. Accumulating data support the role of ERK, JNK and p38 in the pathogenesis of HS^37^. In the present study, KEGG pathway analysis was also performed after pressure treatment. We found that in the 90 days group, metabolic pathways, PI3K-Akt signaling pathway, focal adhesion, apoptosis pathway and the ECM-receptor interaction pathway were enriched with more DEGs, whereas at 120 days group, except for the pathways mentioned above, the AGE-RAGE signaling pathway in diabetic complications, protein digestion and absorption, cAMP signaling pathway were also enriched with DEGs, but the number of DEGs in these pathways was also less than that in the 90 days group. A previous review suggested that the molecular knowledge on the roles of key growth factor pathways in the pathophysiology of hypertrophic scars can explain molecular pathogenesis and provide therapeutic implications^23^. KEGG pathway analysis in our study showed that the outcomes of pressure therapy could also be mediated by a cytokine-cytokine receptor interaction pathway such as the IGF-1/IGF-1R pathway. These results imply that genes associated with the above signaling pathways contributed significantly to the effectiveness of pressure therapy. However, given the currently available information, the association between the mechanisms of pressure therapy and metabolic pathways or focal adhesion, requires further investigation.

In order to establish the accuracy of the results obtained by RNA-seq, we selected seven genes which were proven to be associated with HS pathology. Numerous studies have suggested that the TGF-β/Smad signaling pathway is the key regulator in the pathogenesis of fibrosis via its mediation of cell growth, cell function and extracellular matrix (ECM) deposition^38–40^. Strong evidence has been found that TGF-β1 is up-regulated in keloid tissue, promoting collagen formation and the proliferation and differentiation of dermal fibroblasts^41–43^. Similarly, Sato noted that the TGF-β signaling pathway worked in a combinatorial manner with Wnt/β-catenin signaling^44^, thus co-operation between these two signaling pathways might be relevant to the pathogenesis of hypertrophic scars and keloids. Therefore, we investigated the expression profile of the TGF-β/Smad signaling pathway both by RNA-seq and qPCR. The results revealed that the trend of expression of three genes (TGF-β1, Smad2, Smad3) in the RNA-seq results was consistent with that in the PCR results, indicating that the RNA-seq data were accurate and reliable. In addition, the MAPK signaling pathway was also studied as an important pathway in regulating the progression of HS. Recent studies have demonstrated that ERK and JNK mediate collagen I expression and scarring of rabbit ears, and might be considered as specific drug therapy targets for HS^45^. In our study, the activation of the MAPK signaling pathway was also proposed as a crucial element in the response to pressure therapy. Furthermore, α-SMA is reported to be a crucial factor that contributes to cell contraction, which leads to excessive wound healing and scar contractures^46^. In our study, we found that the expression of α-SMA mRNA was markedly decreased after pressure therapy in the two groups, suggesting that pressure therapy is efficient for HS partly because of the reduction in ECM deposition.

In summary, our results have demonstrated that genes associated with transporter activity and signal transducer activity participated in the treatment of pressure for HS. Furthermore, the RNA-seq results indicate that metabolic and PI3K-Akt signaling pathways are markedly associated with HS regulation in the process of pressure therapy. Therefore, the combination therapy of PI3K-Akt signaling pathways and the development of medicines for regulating metabolic target genes might be considered in clinic. In brief, our findings may provide a promising alternative for therapeutic intervention of specific genes or pathways in the process of pressure therapy for HS.

## Materials and Methods

### Ethics statement

Pig care and housing was carried out in accordance with guidelines established by the Animal Care and Use Committee of China Institute For Radiation Protection. The China Institute For Radiation Protection Animal Care and Use Program is accredited by the Association for the Assessment and Accreditation of Chinese Laboratory Animal Care. Research activities conformed to the statutes of the Animal Welfare Act and guidelines of the Public Health Service as required in the Guide for the Care and Use of Laboratory Animals. The experimental protocols were approved by the Animal Care and Use Committee of the Shanxi Science and Technology Department.

### Animals

Three adolescent female Bama miniature pigs aged 2–3 months and weighing between 21 and 23 kg were used for this study. The Bama minipigs were wild type, without genetic modification. They has been selected by experimental animal breeding, meeting the local standard of Beijing small experimental pig and having high genetic stability. They were purchased from the Beijing Shichuang Century Minipig Breeding Base and raised in the Center for Drug Safety Evaluation, China Institute For Radiation Protection and cared for by an experienced technician. The animals were raised in single cages indoors and provided a laboratory porcine grower diet and water three times per day. They were acclimatized for 2 weeks before initiation of the experiment. Maintenance and killing of the animals followed principles of good laboratory practice in compliance with China national laws and regulations.

### Skin Wounding

The pigs were fasted for 12 hours before surgery. The hair on their backs was clipped and the dorsal skin thoroughly disinfected with betadine solution then rinsed with 75% alcohol. The pigs were pre-medicated with Zoletil (15 mg/kg) by intramuscular injection 10 minutes before skin wounding commenced. When complete anesthesia had been achieved, they were placed in a ventral recumbent position on the operating table^47^. Four deep dermal wounds measuring 8 × 8 cm were created on the back of the pigs in two rows of two columns. An electric dermatome (Tyler Research Corp., Edmonton, AB), set to harvest at a depth of 1.8 mm, was used to strip the deep dermal wounds to the depth of the subcutaneous fat dome sections of the fat cones, although it is known that the actual wound depth obtained with a dermatome is variable. The wounds were denoted left front, left rear, right front or right rear, depending on their position. Bandages were applied after wound hemostasis was achieved, and then removed after 24 hours, the wounds allowed to heal in the open without application of topical agents or dressings. Sixty days after surgery, a homemade elastic knitted device made of leica fabric and designed to exert a pressure of approximately 3.4 kPa on the underlying tissue was applied to the two front wounds. This professional and commercial leica plastic fabric, woven from spandex, is provided by the department of burns and plastic surgery, Taigang General Hospital, where it is used to make pressure suits for scar patients. A sensor was used to measure the pressure once a week to ensure the size was adjusted appropriately.

### Tissue Sampling

At day 90 and 120 post-injury, the animals were anesthetized, and hypertrophic scars were collected from left front (with pressure), left rear (control), and right front (with pressure), right rear (control) on the back of the pigs, respectively. The wounds were down to the subcutaneous fascia using a surgical blade. Each sample was divided into two pieces for molecular analysis, then snap-frozen in liquid nitrogen and stored at −80 °C until processed. The 90 day groups were defined as ‘90d + P’ vs. ‘90d’ and the 120 day groups as ‘120d + P’ vs. ‘120d’ for the RNA-seq analysis.

### RNA extraction and purification

Total RNA was extracted from HS samples using a *mir*Vana^TM^ miRNA isolation kit (Cat#AM1561, Ambion) following the manufacturer’s instructions, the quantity was measured using a NanoDrop Spectrometer (ND-1000 Spectrophotometer, Peqlab). The RNA integrity number (RIN) was tested using an Agilent Bioanalyzer 2100 (Agilent Technologies, Santa Clara, CA, USA). Qualified total RNA was further purified using an RNAClean XP Kit (Cat A63987, Beckman Coulter, Brea, CA, USA) and RNase-free DNase set (Cat#79254, Qiagen GmbH, Germany). The RNA of each sample that passed quality control testing was used for the construction of the library.

### Library preparation and sequencing

Poly-A containing mRNA molecules were purified using poly-T oligo-attached magnetic beads. Following purification, the mRNA was fragmented into small pieces using divalent cations at 94 °C for 8 min. The cleaved RNA fragments were copied into first-strand cDNA using SuperScript II Reverse Transcriptase (Invitrogen, 18064014, Carlsbad, CA, USA) and random primers, followed by second-strand cDNA synthesis using Second Strand Master Mix and isolation using AMPure XP beads (A63881, Beckman). The cDNA fragments then underwent an end repair procedure, a single ‘A’ base was added and they were then ligated to adapters. The products were purified and then enriched using PCR to create the final cDNA library^48^. Purified libraries were quantified using a Qubit® 2.0 Fluorometer (Life Technologies, USA) and validated using an Agilent 2100 bioanalyzer (Agilent Technologies, USA) to confirm insert size and calculate its molar concentration. Clusters were generated using a cBot with the library diluted to 10 pM and sequenced on an Illumina HiSeq 2500 (Illumina, USA). Library construction and sequencing were performed by Shanghai Biotechnology Corporation (Shanghai, China).

### Data analysis for gene expression

Raw reads from the sequenced libraries were preprocessed by the removal of rRNA reads, sequencing adapters, short-fragment reads and other low-quality reads, yielding a dataset consisting of clean reads. Tophat v2.1.0 was used to map the cleaned reads to a pig (*sus scrofa*) reference genome (National Center for Biotechnology Information reference sequence: GCA_000003025.6) allowing for two mismatches^49^.

In order to assess the quality of the sequencing, the gene coverage and sequencing saturation were analyzed. After genome mapping, the open source suite of tools Cufflinks was run with a reference annotation to generate fragments per kilobase of exon per million mapped reads (FPKM) values for standardized calculation of the gene-expression levels^50^. Differentially expressed genes (DEGs) were identified using Cuffdiff software. The calculated gene expression levels could thus be used for comparing gene expression directly between the different samples. The significance threshold of the p-value of multiple tests was set by the false discovery rate (FDR)^51,52^. Fold-change in expression was also estimated according to the FPKM in each sample. Differentially expressed genes were selected using the following filter criteria: FDR ≤ 0.05 and fold-change ≥2.

Gene Ontology (GO) analysis first mapped all DEGs to GO terms within the database (http://www.geneontology.org/) then gene numbers were calculated for every term from the biological process, cellular component and molecular function level domains^53^. Functional enrichment analysis provided the GO terms for the DEGs that were substantially enriched relative to the background in the genome. WEGO^54^ was used to determine the biological function of the DEGs and to plot the distribution of the pig gene functions. In order to provide further insight into the biological functions of the DEGs, pathway assignment analysis of the DEGs was performed based on the Kyoto Encyclopedia of Genes and Genomes (KEGG) database. This analysis identified cell biochemical processes such as metabolism, membrane transport, signal transmission and cell cycle associated with the DEGs that were significantly enriched relative to the entire background of the genome. In the present study, significant pathways were identified as those with P values < 0.05 with the false-discovery rate (FDR) set at <0.05. The Q value indicated the FDR of the specific pathway, lower values indicating greater significance.

### Validation with qRT–PCR

Seven genes associated with HS formation were selected for validation by quantitative PCR (qRT–PCR) using the same RNA samples used in the RNA-seq. Total RNA was extracted and purity calculated from the optical density (OD) ratio (260 nm/280 nm). cDNA synthesis was performed using a ReverTra Ace qPCR RT Kit (Toyobo, Japan) according to the manufacturer’s instructions. All samples were reverse transcribed simultaneously to avoid experimental variation. The qPCR experiments were performed using Power SYBR® Green PCR Master Mix (ABI, USA), templates and primers in a total reaction volume of 10 μL. A 7900 HT Sequence Detection System (ABI, USA) was used with the following cycling parameters: denaturation at 95 °C for 10 min, followed by 40 cycles of 15 s at 95 °C then 1 min at 60 °C. The densitometric values for scar samples treated with pressure were compared with those without treatment to determine the relative expression levels at each timepoint. The results from three independent reactions were used to determine the relative expression levels of the target genes, which were normalized against the expression level of succinate dehydrogenase (SDHA) using the 2^−ΔΔCt^ method. The gene expression levels for scars treated with pressure were compared with those of untreated scars based on the cDNA sequencing results. All mRNA primers were designed and synthesized by Sangon Biotech (Shanghai, Co., Ltd.). The primer sequences (*sus scrofa*) used for gene amplification were as described in Table S1.

### Statistical analysis

All data were analyzed by analysis of variance (ANOVA) using SPSS 16.0 software (SPSS Inc., Chicago, IL, USA) with data expressed as mean ± SD. Comparisons among the groups were calculated using one-way ANOVA. The differences among treatment means were evaluated with a Duncan’s multiple range test at a 0.05 probability level. Figures were generated using OriginPro 8.5 (OriginLab Corp., Northampton, MA, USA).

### Data availability

The datasets generated in the course of the current study are available from the corresponding author on reasonable request.

## Electronic supplementary material

Table S1
