# Supplementary material for: RNA-seq-based analysis of the hypertrophic scarring with and without pressure therapy in a Bama minipig model
Source: Sci Rep. 2018 Aug 7;8:11831. doi: 10.1038/s41598-018-29840-6 (PMC6081447; doi:10.1038/s41598-018-29840-6)
Supplement: Supplementary file 1 — Table S1 [file 41598_2018_29840_MOESM1_ESM.docx]

RNA-seq-based analysis of the hypertrophic scarring with and without pressure therapy in a Bama minipig model

Baimei Liu^1,2,3^, Yang Liu^1,2,3^, Li Wang^1,2,3^, Chunsheng Hou^4^, and Meiwen An^1,2,3*^

**Table S1. Primers used for real-time quantitative RT-PCR.**

| Gene name | Gene description | Genebank No. | Primer Sequence (5' to 3') | |
| --- | --- | --- | --- | --- |
| TGF-β1 | transforming growth factor beta 1 | 397078 | F | CTCCGATTTAACCCCTAGCC |
|  |  |  | R | TGGGCTTCCTTTTCCTTTCT |
| Smad-2 | SMAD family member 2 | 100155304 | F | GGCTCAGTCCGTTAATCAGG |
|  |  |  | R | CTGCCTTCGGTATTCTGCTC |
| Smad-3 | SMAD family member 3 | 397260 | F | TCCATGAAGGAAGAGGCAGT |
|  |  |  | R | CTGAGCATGTGAGAATGTGGA |
| JNK | c-jun N-terminal kinase | 396610 | F | TGCCATTCCTTCTTCATTCC |
|  |  |  | R | TGCCCCATTTTGTTTTGTCT |
| p38 | p38 MAP kinase | 100156630 | F | GCCAAGGTGTCTCCATTTCT |
|  |  |  | R | CTTCCTTCTCGCTCCAGTTG |
| FN1 | fibronectin 1 | 397620 | F | AGACCCCATCCCAGTTTGTT |
|  |  |  | R | TGATGCTTGGAGAAGCTGTG |
| α-SMA | Alpha-smooth muscle actin | 733615 | F | CGCAAATACTCCGTCTGGAT |
|  |  |  | R | GGCTTCGTCGTACTCCTGTT |
| SDHA | succinate dehydrogenase complex flavoprotein subunit A | 780433 | F | CACACGTTGTACGGAAGGTCT |
|  |  |  | R | ATCAGGAGATCCAAGGCAAA |
